# Crohn’s Disease as a Possible Risk Factor for Failed Healing in Ileocolic Anastomoses

**DOI:** 10.3390/jcm12082805

**Published:** 2023-04-11

**Authors:** Julian Thomas Schweer, Philipp-Alexander Neumann, Philipp Doebler, Anna Doebler, Andreas Pascher, Rudolf Mennigen, Emile Rijcken

**Affiliations:** 1 Department of General, Visceral, and Transplantation Surgery, Muenster University Hospital, 48149 Muenster, Germany; 2 Department of Surgery, Klinikum rechts der Isar, Technical University of Munich School of Medicine, 81675 Munich, Germany; 3 Department of Statistics, Chair of Statistical Methods in Social Sciences, Technical University of Dortmund, 44227 Dortmund, Germany; 4 Psychological Assessment and Methods Group, Institute of Psychology, Faculty of Educational Sciences, University of Duisburg-Essen, 45141 Essen, Germany; 5 Medizinisches Versorgungszentrum Portal 10, 48155 Muenster, Germany

**Keywords:** Crohn’s disease, anastomotic healing, surgery, ileocolic anastomosis, risk factors

## Abstract

Anastomotic leakage (AL) after colorectal resections is a serious complication in abdominal surgery. Especially in patients with Crohn’s disease (CD), devastating courses are observed. Various risk factors for the failure of anastomotic healing have been identified; however, whether CD itself is independently associated with anastomotic complications still remains to be validated. A retrospective analysis of a single-institution inflammatory bowel disease (IBD) database was conducted. Only patients with elective surgery and ileocolic anastomoses were included. Patients with emergency surgery, more than one anastomosis, or protective ileostomies were excluded. For the investigation of the effect of CD on AL 141, patients with CD-type L1, B1–3 were compared to 141 patients with ileocolic anastomoses for other indications. Univariate statistics and multivariate analysis with logistic regression and backward stepwise elimination were performed. CD patients had a non-significant higher percentage of AL compared to non-IBD patients (12% vs. 5%, *p* = 0.053); although, the two samples differed in terms of age, body mass index (BMI), Charlson comorbidity index (CCI), and other clinical variables. However, Akaike information criterion (AIC)-based stepwise logistic regression identified CD as a factor for impaired anastomotic healing (final model: *p* = 0.027, OR: 17.043, CI: 1.703–257.992). Additionally, a CCI ≥ 2 (*p* = 0.010) and abscesses (*p* = 0.038) increased the disease risk. The alternative point estimate for CD as a risk factor for AL based on propensity score weighting also resulted in an increased risk, albeit lower (*p* = 0.005, OR 7.36, CI 1.82–29.71). CD might bear a disease-specific risk for the impaired healing of ileocolic anastomoses. CD patients are prone to postoperative complications, even in absence of other risk factors, and might benefit from treatment in dedicated centers.

## 1. Introduction

The failure of anastomotic healing is a major concern in gastrointestinal surgery. In recent studies, the rate of anastomotic leakage (AL) after colorectal resections still ranges from 4 to 11% [1,2,3,4]. Especially in Crohn’s disease (CD), the failure of anastomotic healing may lead to a detrimental postoperative course. In these specific patients, the rates of AL vary from 3–23% [5,6,7,8]. The failure of anastomotic healing can become evident as a leakage occurring at the anastomotic site with septic complications, such as peritonitis or intra-abdominal abscesses. The chronic failure of anastomotic healing can result in the development of entero-cutaneous fistulas at incisions sites or drainage canals, sometimes occurring within weeks after being discharged from the hospital. Thus, anastomotic complications after colorectal resections have a substantial negative impact on the postoperative quality of life [9]. Moreover, the occurrence of postoperative intraabdominal septic complications in CD is associated with an increased risk of surgical recurrence [10]. A wide variety of risk factors for failed anastomotic healing after colorectal resections have been described, such as obesity, male gender, deep colorectal anastomoses, preoperative radio-chemotherapy, the Charlson comorbidity index (CCI) of a score of two or higher, an American Society of Anesthesiologists (ASA) score ≥ 3, anticoagulant treatment, low serum albumin levels, and intra-operative complications [2,4,11]. Furthermore, numerous risk factors have been described, in particular in patients with CD. Poor nutritional status, pre-operative anemia, hypoalbuminemia, immunosuppression, previous intestinal resection, smoking, a penetrating type of disease, recurrent CD, inflammation in histological margins, and a disease duration of over 10 years were found to have a negative influence on postoperative intra-abdominal septic complications after an ileocolic resection for CD [7,12,13,14,15,16,17,18,19,20]. Additionally, the long-term use of highly dosed steroids, azathioprine, and the presence of pre-operative abscesses or fistulas were associated with higher rates of anastomotic complications [5,12,15,16,21]. The role of anti-inflammatory and immunomodulating medications in septic complications after surgery for CD remains controversial: some studies identified anti-tumor necrosis factor-alpha (anti-TNF-α) agents as a risk factor for postoperative complications in CD, such as wound infections, intra-abdominal abscesses, and leakages, whereas others did not [7,16,22,23]. However, it remains unclear whether AL is increased in patients with CD compared to other colorectal diseases. Additionally, if so, it is not known whether CD itself contributes to the failure of anastomotic healing or if this is caused by other risk factors, which are often concomitant in these patients. 

In the present study, we especially inquired whether there is a disease-specific risk for failed anastomotic healing in patients with CD. We only observed elective ileocolic resections with anastomosis since this is the typical procedure for CD.

## 2. Materials and Methods

The study was conducted according to the guidelines of the Declaration of Helsinki. The ethical review and approval were waived for this study, due to its anonymized and retrospective design.

The study was conducted at a tertiary referral center for inflammatory bowel diseases. The department’s prospectively maintained and anonymized inflammatory bowel disease database was reviewed to identify all consecutive patients who had undergone an elective ileocolic anastomosis for CD-type L1, B1–3 according to the Montreal classification during the period of January 2004 to December 2011. These patients were compared to consecutive patients with elective ileocolic anastomosis for conditions other than CD at the same institution during the same period. Only patients with single ileocolic anastomosis were included. Patients with more than one anastomosis or stricturoplasties and patients receiving a diverting ileostomy were excluded, as were patients with ileocolic resections without anastomosis. Patients with free perforations and systemic sepsis being emergency cases were also excluded. 

Medical records were reviewed for peri-operative data on sex, age, body mass index (BMI), comorbidities, ASA score, CCI, nicotine use, diabetes mellitus, liver diseases, peri-operative immunomodulating medication, steroids, anticoagulants, previous minor or major intra-abdominal surgeries, type of operation, laparoscopic or conventional surgery, type of anastomosis, hand-sewn or stapled anastomoses, surgeon’s experience, pre-operative laboratory parameter (hemoglobin, leukocyte counts, C-reactive protein (CRP)), pre-existent intra-abdominal abscesses and fistula, occurrence of AL or fistula, surgical-site infections, re-operation rates, and mortality, and additionally in CD: duration of disease and extra-intestinal manifestations. Since pre-operative serum albumin-levels were not available for all patients, the BMI was measured as a surrogate marker for their nutritional status. 

All patients had prophylactic single-shot antibiotics with cefuroxime and metronidazole during the induction of narcosis. Drains were not used for routine ileocolic anastomoses. Pre-operative mechanical bowel preparation was not conducted. There was no standardized enhanced recovery after surgery (ERAS) program; however, the patients were treated according to the standard operating procedures of our department. 

AL was defined as any postoperative bowel leak at the site of the ileocolic anastomosis with fecal or purulent discharge leading to peri-anastomotic abscesses, peritonitis, or entero-cutaneous fistulas within 90 days after surgery. AL was suspected when clinical symptoms, such as fever, bowel paralysis, or abdominal defense, were observed. The clinical work-up, in order to rule out AL, included a physical examination, laboratory parameter, computed tomography (CT)-imaging, and endoscopy. All patients suspected of having AL were re-operated on, confirming the diagnosis of failed anastomotic healing intra-operatively. 

Surgical-site infections (SSIs) were defined as superficial when an infection occurred within 90 days after the operation and the infection involved only the skin or subcutaneous tissues of the incision site itself, as deep when the infection involved deep soft tissues as fascial and muscle layers of the incision site, and as organ/space when the infection involved any part of the anatomy other than the incision, which was opened or manipulated during an operation [24,25]. Wound infections were opened at the bedside or during surgery. The CCI reflects the number, severity, and interaction of individual comorbidities [26].

The results are presented as mean values ± standard deviation or medians and range where indicated. A pre-study power calculation was not performed. In the first step, two cohorts were compared to each other for demographics and clinical parameters univariately. Second, the parameters associated with AL were analyzed for both groups together, first by univariate tests and then multivariate analysis. In order to evaluate the risk factors in patients with CD more specifically, a separate analysis was performed solely in the CD cohort as well. Univariate statistical evaluations were performed using Fisher’s exact test for categorical variables and proportions and the *t*-test with Welch’s approximation to degrees of freedom for continuous variables using R 4.1.2. [27]. Multivariate analysis was performed using a logistic regression with R 4.1.2 [27]. The initial model included all risk factors, which were also used in the univariate analyses; however, the non-relevant risk factors were eliminated with the help of a stepwise backward search using the Akaike information criterion (AIC) and the more conservative Bayesian information criterion (BIC). For an alternative point estimate for CD as a risk factor for AL, a matching procedure based on a generalized boosted logistic regression for the calculation of propensity scores was implemented [28] using the R-package twang [29]. This method reweights the control sample so that the demographic and clinical variables are comparable. Matching was performed for sex, age, BMI, CCI, leukocyte count, hemoglobin level, type of primary surgeon, stapler vs. hand-sewn, and type of anastomosis. *p*-values of < 0.05 were considered significant in the univariate analyses. For the stepwise procedures, we reported the *p*-values; however, we were cautious that these are conditional to the final model.

## 3. Results

A total of 141 patients with ileocolic anastomosis for CD were compared to 141 patients receiving ileocolic anastomosis for other reasons (Table 1). 

For CD, 110 patients underwent an elective ileocolic or right hemicolic resection for the medically refractory stenosis of the terminal ileum or penetrating disease with intra-abdominal abscesses or blind-ending fistulas, 29 patients had a resection of a previous ileocolic anastomosis for surgical recurrence, and 2 patients underwent the elective restoration of intestinal continuity after a previous surgery with a complicated postoperative course and take down of anastomosis. In the controls, the indications for elective surgery were: tumor (malignant: *n* = 88, benign: *n* = 36, neuroendocrine tumor: *n* = 11), recurrent intestinal bleeding (*n* = 5), or the restoration of intestinal continuity (*n* = 1). Demographic details can be observed in Table 1. The mesentery was divided close to the bowel wall (tubular) for a benign disease and oncologically (centrally) at the vessels’ origin for a malignant disease. Since abscesses were small and a part of the inflammatory mass, no pre-operative interventional drainage was possible. Laparoscopy was performed on 78 patients in CD and 38 in controls with 10.3% vs. 7.9% conversions. The reasons for the conversion were extensive adhesions (*n* = 4), abscesses (*n* = 2), or both (*n* = 2) in CD and unclear intra-operative findings (*n* = 1), unclear intra-operative findings and extensive adhesions (*n* = 1), or extensive meteorism (*n* = 1) in the controls. There were no robotic procedures. The surgical details can be observed in Table 2. 

Surgical complications were assessed according to the Clavien–Dindo classification [30] (Table 3). The reasons for re-operations were anastomotic leakage (17 CD vs. 7 control), small bowel perforation (0 vs. 1), ileus (2 vs. 6), bleeding (1 vs. 1), fascial dehiscence (5 vs. 6), surgical wound-revision (4 vs. 2), planned 2nd-look laparotomy (0 vs. 2), or unclear abdominal pain (2 vs. 0) (re-operations according to Clavien–Dindo-type IIIb; patients can have more than one reason for a re-operation). Anastomotic leakage occurred in 17 patients (12.1%) in CD vs. 7 patients (4.4%) in the controls. Among these, there were entero-cutaneous fistulas (3 in the CD group and 1 in the control group).

The univariate analysis (Table 4) revealed that among the 24 patients with failed anastomotic healing, CD tended to be more prevalent; however, this was not statistically significant (17:7 vs. 124:134, *p* = 0.053). In this analysis, age (*p* = 0.022), a pre-operative use of steroids > 20 mg/d (*p* = 0.030), pre-existing abdominal sepsis (*p* = 0.04), fistulas (*p* = 0.010), or abscesses (*p* = 0.005) were shown to be associated with the failed healing of ileocolic anastomoses. Pre-operative CRP was increased in patients with failed healing (*p* = 0.036). 

AIC-based stepwise logistic regression identified CD as a factor for impaired anastomotic healing (final model: *p* = 0.027, OR: 17.043, CI: 1.703–257.992). Additionally, a CCI of 2 or higher (*p* = 0.010) and abscesses (*p* = 0.038) increased the risk of disease (full model output in Table 5). The alternative point estimate for CD as a risk factor for AL based on the propensity score weighting also resulted in an increase in risk, albeit a lower one (*p* = 0.005, OR 7.36, CI 1.82–29.71). The more conservative BIC-based selection only identified abscesses as a risk factor (*p* = 0.003, OR = 4.054, CI = 1.526–10.129) for AL. 

The cohort of CD patients (*n* = 141) was analyzed again, separately, in order to identify further risk factors for AL in CD, making the cohort comparable to other studies observing AL in CD only. In this univariate analysis (Table 6), pre-existing intra-abdominal abscesses (*p* = 0.004), increased pre-operative CRP (*p* = 0.035), and low hemoglobin levels (*p* = 0.027) were found to be associated with impaired anastomotic healing. 

In the cohort of CD patients, AIC-based stepwise logistic regression identified pre-existing abdominal abscesses and high BMI levels as the factors for an increased risk for anastomotic leakage, while operations by consultants were found to decrease the risk level. The results are depicted in Table 7. The more conservative BIC-based stepwise logistic regression presented fewer risk factors; however, again pre-existing abdominal abscesses were observed to be a risk factor for anastomotic leakage in CD patients (final model: *p* = 0.001, OR: 7.552, CI: 2.304–27.124), whereas operations performed by consultants were protective (final model: *p* = 0.026, OR: 0.252, CI: 0.070–0.843).

## 4. Discussion

By comparing CD patients with those having undergone an ileocolic anastomosis for other reasons, CD was identified as a disease-specific risk factor for impaired anastomotic healing. Although this might be expected by the inflammatory and penetrating nature of the disease, studies, to date, have failed to prove CD as an independent risk factor. However, the topic is still discussed, controversially. By using modern and more sensible statistical methods, further insights might be achieved, even in smaller cohorts. 

Most studies overlooking the postoperative complications after colorectal surgery are limited by a heterogeneous cohort of patients with different types of anastomoses, different underlying diseases, a wide variety of comorbidities and medications, and different definitions of anastomotic leakage [31]. In the present study, we restricted the analysis to one, single type of anastomosis, which is typically applied in CD. Patients with more than one anastomosis, emergency surgery, or protective ileostomies were rigorously excluded, thereby eliminating both unequally distributed detrimental and protective factors. We also considered typical postoperative complications for CD, such as entero-cutaneous fistulas deriving from anastomosis as the failure of anastomotic healing, even if they appeared after the patient’s discharge from the hospital. Late-healing failures can even occur over 30 days after the primary surgery and are often not included in complications statistics [32]. Our observation period of 90 days was therefore comparatively longer than that in other studies [2,11,12,15,20,33]. 

However, our study was clearly limited by its retrospective nature and the wide variety of indications for ileocolic anastomoses in the control group. Unfortunately, the data on nutritional state, other than BMI, were not consistently available and were therefore not included in the analysis. CD patients always differ from the controls in many variables, and the estimation of the independent effect of CD must be elucidated by multivariate statistical methods. To be cautious, we included the very conservative BIC for the logistic regression with a backward stepwise elimination. In contrast to the AIC-based stepwise regression and propensity score-weighted analysis, the BIC-based analysis attributed the risk of AL solely to pre-existing abdominal abscesses. 

No randomized trial can be designed to clarify our main research question. Matched-pair cohort analyses would not be applicable if age or malignancies are considered for the matching criteria, since not many young patients have ileocolic anastomoses for reasons other than CD. Similarly, neither immunomodulators nor high-dose steroids could be considered in a matched-pair design, since both were rare in the controls. In the future, increasing the data pool by large register studies could strengthen our findings further.

As expected, there were striking differences between the CD group and controls. Various factors regarded as protective for anastomotic healing, such as a younger age, lower BMI, low CCI, benign disease, or operations by experienced surgeons were more present in the CD group. Immunomodulators and high-dose steroids were more frequent in the CD group, whereas anticoagulants, diabetes, or liver diseases were more prevalent in the controls. Though presumed protective factors were increased in the CD group, there were substantially more septic complications, especially a higher incidence of anastomotic complications. This is in line with the clinical experience of many surgeons. Therefore, the question remains whether there is a disease-specific risk for surgical complications after colorectal resections. At least for incisional SSI, CD has been shown to be an independent risk factor, as shown by various authors [34,35,36]. Moreover, a significantly increased risk for catheter-associated bloodstream infections after bowel resections was observed in patients with CD [37]. Additionally, van Arendonk et al. found in a large register study observing left hemi- and subtotal colectomies a higher in-hospital mortality rate, a higher rate of complications, and a higher rate of ostomies in patients with IBD, when compared to colon cancer or diverticular disease [38]. Additionally, using the ACS-NSQIP database, Larson et al. demonstrated more frequent surgical complications, sepsis, and unplanned readmissions after ileocolic anastomosis in patients with CD compared to patients with right-sided colon cancer [19]. Furthermore, Lipska et al. found in their univariate analysis an increased risk for anastomotic leakage after colorectal anastomoses in patients with CD; however, this was not confirmed in the multivariate analysis, not using AIC-based stepwise logistic regression [39].

These findings might raise the question whether CD patients should preferably be treated in centers specially dedication to inflammatory bowel diseases. Studies based on the nationwide inpatient sample revealed lower postoperative in-hospital mortality rates in high-volume hospitals in patients with CD compared to non-high-volume hospitals [40,41,42].

A single surgeon, but also institutional experience, has an impact on postoperative outcomes. The postoperative outcome is not only a result of the operation itself, but also of pre-operative optimization and postoperative care. This might be of particular relevance to patients with CD, since they often present with complex diseases and specific medications. We observed the factor surgeon by analyzing consultants and residents separately. In the multivariate analysis, but surprisingly not in the univariate analysis, a reduced risk of AL was observed when the surgeon was more experienced (consultant); although, anastomoses in the resident group were all supervised. This might be the result of a statistical approach; however, the safety of anastomoses by trainees was controversially discussed in the literature with increased operative times and morbidity rates when residents were involved. Nonetheless, operations performed by residents under supervision are unanimously believed to be safe [43,44]. However, the better results for consultants in our data might underline the complexity of surgery for CD.

Several studies have identified the risk factors for anastomotic leakage after lower intestinal resections in general and in CD specifically. In the present study, some of these factors could be confirmed; however, others were not. Steroids > 20 mg/d is consistently reported as a risk factor for anastomotic leakage in CD [5,12,16]. Other immunomodulators, especially anti-TNF-α agents, were not found to have a negative impact on anastomotic healing in our study. This can be explained by a policy of discontinuation of biologicals for at least four weeks before elective surgery for CD in our institution, covering approximately two half-life times of most biological factors. Some meta-analyses and population-based studies found anti-TNF-α agents to be a risk factor for septic complications after surgery in CD [18,22,23]; however, most studies on this topic lack homogeneous doses and intervals of these medications before surgery. The measurement of anti-TNF-α-levels in patient’s serum samples might allow a more adequate assessment of postoperative risks when using anti-TNF-α agents [45]. Additionally, low levels of hemoglobin and elevated CRP levels before surgery were found to be associated with failed anastomotic healing in CD [15,18,46]. In agreement with the other studies, we found that pre-existing intra-abdominal sepsis is a strong predictor for impaired anastomotic healing in CD [5,12,16,33]. 

If CD itself is independently associated with a higher risk of impaired anastomotic healing, one might look for disease-related molecular disturbances of tissue healing. Indeed, many inflammatory mediators, such as TNF-α, interleukins, adhesion molecules, growth factors, or matrix metalloproteinases, involved in anastomotic healing in the gastrointestinal tract are also key mediators in inflammatory processes in CD [47] However, explanations for molecular mechanisms cannot be drawn from this study. However, as a speculation, CD can be looked at as a disturbed immunological response to inflammatory processes leading to ongoing inflammation with tissue damage. This inadequate immune response might also impair mechanisms of anastomotic healing, which follow comparable patterns of tissue damage, inflammatory response, proliferation, and remodeling. Interestingly, the role of endoluminal bacteria for anastomotic healing is still debated [1,8] and the composition of microbiota might be altered in CD, too [8,48]. 

The role of mesenteric adipose tissue (MAT) in CD remains a matter of debate. Although there is evidence that MAT is related to the recurrence of Crohn’s disease, it could not be shown that MAT is associated with a higher risk of anastomotic leakage at present [8].

## 5. Conclusions

In our model, a disease-specific risk for the impaired healing of ileocolic anastomoses was observed in patients with CD. This indicated that CD patients should be observed as patients at special risk for postoperative anastomotic complications, even if other risk factors are absent.

## Figures and Tables

**Table 1:** Demographics.

	CD, *n* = 141, (%)	No CD, *n* = 141, (%)	*p*-Value
Sex [m:f]	48 (34%):93 (66%)	74 (52.5%):67 (47.5%)	**0.003 ^#^**
Age [years]	35.3 ± 11.9	64.0 ± 12.2	**<0.001 ^#^**
BMI [kg/m^2^]	21.8 ± 4.0	26.4 ± 4.8	**<0.001 ^#^**
ASA classification			**<0.001 ^#^**
I	24 (17%)	16 (11.3%)	
II	97 (68.8%)	73 (51.8%)	
III	20 (14.2%)	46 (32.6%)	
IV	0 (0%)	6 (4.3%)	
CCI [points]			**<0.001 ^#^**
0–1	138 (97.9%)	52 (36.9%)	
≥2	3 (2.1%)	89 (63.1%)	
Type of disease:			**<0.001 ^#^**
Crohn’s	141 (100%)	0 (0%)	
Carcinoma/NET	1 (0.7%) ^§^	99 (70.2%)	
Adenoma	0 (0%)	36 (25.5%)	
Stenosis	102 (72.3%)	0 (0%)	
GI bleeding	0 (0%)	5 (3.5%)	
Duration of CD [years]	9.9 ± 8.5	n.a.	**—**
Extra-intestinal manifestations of CD	15 (10.6%)	n.a.	**—**
Pre-existing intra-abdominal abscesses or fistula	55 (39%)	13 (9.2%)	**<0.001 ^#^**
Smoking	41 (29.1%)	17 (12.1%)	**<0.001 ^#^**
Steroids > 20 mg/d	28 (19.9%)	2 (1.4%)	**<0.001 ^#^**
Azathioprine/5- MCP	46 (32.6%)	1 (0.7%)	**<0.001 ^#^**
Anti-TNF-α (4–8 weeks prior to surgery)	31 (22%)	0 (0%)	**<0.001 ^#^**
Other immunomodulators ^+^	4 (2.8%)	4 (2.8%)	1.000 ^#^
Anticoagulation:			**<0.001 ^#^**
none	141 (100%)	106 (75.2%)	
Acetylsalicylic acid (ASA)	0 (0%)	23 (16.3%)	
Coumarins	0 (0%)	12 (8.5%)	
Diabetes mellitus	2 (1.4%)	20 (14.2%)	**<0.001 ^#^**
Liver disease	3 (2.2%)	6 (4.3%)	0.15 ^#^
Previous abdominal surgery:			0.28 ^#^
None	67 (47.5%)	80 (56.8%)	
Minor	26 (18.4%)	26 (18.4%)	
Major	48 (34%)	35 (24.8%)	
CRP [mg/L] **	6.1 ± 7.8	2.7 ± 4.1	0.07 *
Leukocyte count [1000/µL] **	9.0 ± 4.2	8.1 ± 3.9	0.18 *
Hemoglobin level [g/dL] **	12.3 ±1.9	12.2 ± 2.3	0.82 *

* *t*-test; ^#^ Fisher’s exact test; ** laboratory parameter on the day of admission; ^§^ Crohn’s disease-associated carcinoma; ^+^ cyclosporine, tacrolimus, methotrexate, mycophenolate mofetil; 5-MCP: 5-mercaptopurine; anti-TNF-α: anti-tumor necrosis factor-alpha therapy; ASA: acetylsalicylic acid ASA classification: American Society of Anesthesiologists (ASA) Physical Status classification system; BMI: body mass index; CCI: Charlson comorbidity index; CD: Crohn’s disease; CRP: C-reactive protein; GI: gastrointestinal; n.a.: not applicable; NET: neuroendocrine tumor.

**Table 2:** Surgical details.

	CD, *n* = 141, (%)	No CD, *n* = 141, (%)	*p*-Value
Procedure:			**<0.001 ^#^**
Right hemicolectomy	12 (8.5%)	103 (73.1%)	
Ileocolic resection	98 (69.5%)	37 (26,2%)	
Anastomotic resection	29 (20.6%)	0 (0%)	
Restoration of continuity	2 (1.4%)	1 (0.7%)	
Access to the abdomen:			**<0.001 ^#^**
Laparoscopic	70 (49.6%)	35 (24.8%)	
Converted	8 (5.7%)	3 (2.1%)	
Open	63 (44.7%)	103 (73.1%)	
Type of anastomosis:			**<0.001 ^#^**
End-to-end	51 (36.2%)	86 (61,0%)	
Side-to-side	89 (63.1%)	49 (34.8%)	
End-to-side	1 (0.7%)	3 (2.1%)	
Side-to-end	0 (0%)	3 (2.1%)	
Stapled:handsewn	78 (55.3%):63 (44.7%)	23 (16.3%):118 (83.7%)	**<0.001 ^#^**
Type of primary surgeon:			**0.002 ^#^**
Consultant	105 (74.5%)	80 (56.7%)	
Resident	36 (25.5%)	61 (43.3%)	

^#^ Fisher’s exact test; CD: Crohn’s disease; SD: standard deviation.

**Table 3:** Surgical outcomes and complications.

	CD, *n* = 141, (%)	No CD, *n* = 141, (%)	*p*-Value
Clavien–Dindo [grade]			
0 (no complications)	74 (52.5%)	71 (50.4%)	0.81 ^#^
I	24 (17%)	21 (14.9%)	0.74 ^#^
II	19 (13.5%)	17 (12.1%)	0.18 ^#^
IIIa	1 (0.7%)	1 (0.7%)	1.00 ^#^
IIIb (re-operations):	20 (14.2%)	22 (15.6%)	0.87 ^#^
Anastomotic leakage	17 (12.1%)	7 (5.0%)	0.052 ^#^
Ileus	2 (1.4%)	6 (4.3%)	0.28 ^#^
Fascia dehiscence	5 (3.5%)	5 (3.5%)	1.00 ^#^
IVa	1 (0.7%)	3 (2.1%)	0.62 ^#^
IVb	2 (1.4%)	3 (2.1%)	1.00 ^#^
SSI ^§^:			
Superficial	35 (24.8%)	24 (17.0%)	0.14 ^#^
Deep	16 (11.3%)	10 (6.2%)	0.30 ^#^
Organ/space	7 (4.9%)	9 (5.6%)	0.80 ^#^
Hospital stay [mean ± SD]	13.7 ± 17.3	14.5 ± 11.9	0.564 *

* *t*-test; ^#^ Fisher’s exact test; ^§^ patient can have multiple listings for surgical-site infections (SSIs) since superficial, deep, and organ/space SSIs can occur within the same patient; CD: Crohn’s disease; SSI: surgical-site infection.

**Table 4:** Univariate analysis of anastomotic leakage (all patients).

	Failed Healing, *n* = 24, (8.5%)	Regular Healing, *n* = 258, (91.5%)	*p*-Value
Sex [m:f]	11 (45.8%):13 (54.2%)	111 (43%):147 (57%)	0.83 ^#^
Age [years]	40.8 ± 18.7	50.4 ± 18.6	0.022 *
BMI [kg/m^2^]	24.3 ± 4.3	24.1 ± 5.0	0.84 *
CCI [points]			0.82 ^#^
0–1	17 (70.8%)	173 (65.7%)	
≥2	7 (29.2%)	85 (34.3%)	
ASA classification			0.28 ^#^
I	1 (4.2%)	39 (15.1%)	
II	19 (79.2%)	150 (58.1%)	
III	4 (16.7%)	62 (24.0%)	
IV	0 (0%)	6 (2.3%)	
N/A	0 (0%)	1 (0.4%)	
CD [yes/no]	17 (70.8%):7 (29.2%)	124 (48.1%):134 (51.9%)	0.053 ^#^
Carcinoma/NET			0.56 ^#^
no	18 (75%)	164 (63.6%)	
yes	6 (25%)	94 (36.4%)	
Smoking	7 (29.2%)	51 (19.8%)	0.29 ^#^
Steroids > 20 mg/d	6 (25%)	24 (9.3%)	**0.030 ^#^**
Anti-TNF-α 4–8 weeks prior to surgery	3 (12.5%)	28 (10.9%)	0.74 ^#^
Azathioprine/5-MCP	5 (20.8%)	42 (16.3%)	0.57 ^#^
Other immunomodulators ^+^	1 (4.2%)	5 (1.9%)	0.42 ^#^
Anticoagulation			1.00 ^#^
None	21 (87.5%)	225 (87.2%)	
ASA	2 (8.3%)	22 (8.5%)	
Coumarins (±ASA)	1 (4.2%)	11 (4.3%)	
Previous abdominal surgery			0.58 ^#^
None	12 (50%)	135 (50.0%)	
Minor	3 (12.5%)	49 (12.5%)	
Major	9 (37.5%)	74 (37.5%)	
Pre-existing intra-abdominal sepsis	10 (41.7%)	56 (21.7%)	**0.04 ^#^**
Pre-existing intra-abdominal fistula	9 (37.5%)	39 (15.1%)	**0.01 ^#^**
Pre-existing intra-abdominal abscess	8 (33.3%)	28 (10.9%)	**0.005 ^#^**
Liver disease	1 (4.2%)	8 (3.1%)	0.56 ^#^
Diabetes mellitus	2 (8.3%)	20 (7.2%)	1.00 ^#^
CRP [mg/L] **	11.1 ± 10.4	3.5 ± 5.2	0.036 *
Leukocyte count [1000/µL] **	10.0 ± 4.7	8.4 ± 4.0	0.13 *
Hemoglobin level [g/dL] **	11.8 ± 1.8	12.3 ± 2.1	0.18 *
Type of operation			0.86 ^#^
Right hemicolectomy	9 (37.5%)	106 (41.1%)	
Ileocecal resection	12 (50%)	123 (47.7%)	
Anastomotic resection	3 (12.5%)	26 (10.1%)	
Restoration of continuity	0 (0%)	3 (1.2%)	
Access to the abdomen			0.48 ^#^
Laparoscopic	7 (29.2%)	98 (38.0%)	
Converted	0 (0%)	11 (4.3%)	
Open	17 (70.8%)	149 (57.8%)	
Type of surgeon			0.50 ^#^
Resident	10 (41.7%)	87 (33.7%)	
Consultant	14 (58.3%)	171 (66.3%)	
Stapled:handsewn	9 (37.5%):15 (62.5%)	92 (35.7%):166 (64.3%)	0.83 ^#^
Type of anastomosis			0.17 ^#^
End-to-end	11 (45.8%)	126 (48.8%)	
Side-to-side	11 (45.8%)	127 (49.2%)	
End-to-side	1 (4.2%)	3 (1.2%)	
Side-to-end	1 (4.2%)	2 (0.8%)	

* *t*-test; ^#^ Fisher’s exact test; ** laboratory parameter on the day of admission; ^+^ cyclosporine, tacrolimus, methotrexate, mycophenolate mofetil; 5-MCP: 5-mercaptopurine; ASA: acetylsalicylic acid; anti-TNF-α: anti-tumor necrosis factor-alpha therapy; ASA classification: American Society of Anesthesiologists (ASA) Physical Status classification system; BMI: body mass index; CCI: Charlson comorbidity index; CD: Crohn’s disease; CRP: C-reactive protein; GI: gastrointestinal; NET: neuroendocrine tumor.

**Table 5:** Final logistic regression model after Akaike information criterion (AIC)-based forward variable selection. *p*-values are conditional on the final model specification.

	OR *	95% CI **	*p*-Value
Crohn’s disease	17.0	1.70–258	**0.027**
CCI [points]			
0–1	—	—	
≥2	21.9	2.59–294	**0.010**
Type of surgeon			
Resident	—	—	
Consultant	0.40	0.14–1.12	0.082
Pre-existing intra-abdominal abscess	3.66	1.05–12.5	**0.038**
Access to the abdomen			
Laparoscopic or converted	—	—	
Open	2.44	0.83–7.77	0.114
Stapled vs. handsewn			
Handsewn	—	—	
Stapled	4.01	0.67–37.0	0.167
Type of anastomosis			
End-to-end	—	—	
End-to-side	13.3	0.51–191	0.062
End-to-end	0.27	0.03–1.38	0.163
Side-to-end	152	2.66–9.815	**0.013**
Age [years]	0.96	0.91–1.00	0.060
BMI [kg/m^2^]	1.08	0.98–1.19	0.120

* OR = odds ratio; ** CI = confidence interval, BMI: body mass index; CCI: Charlson comorbidity index.

**Table 6:** Univariate analysis of anastomotic leakage in Crohn’s disease only.

	Failed Healing in CD, *n* = 17 (12.1%)	Regular Healing in CD, *n* = 124 (87.9%)	*p*-Value
Sex [m:f]	7 (41.2%):10 (58.8%)	41 (33.1%):83 (66.9%)	0.59 ^#^
Age [years]	31.4 ± 10.2	35.8 ± 12.1	0.12 *
BMI [kg/m^2^]	23.5 ± 4.4	21.6 ± 3.9	0.11 *
CCI [points]			0.32 ^#^
0–1	16 (94.1%)	122 (98.4%)	
≥2	71 (5.9%)	2 (1.6%)	
ASA classification			0.38 ^#^
I	1 (5.9%)	23 (18.5%)	
II	14 (82.4%)	82 (66.1%)	
III	2 (11.8%)	18 (14.5%)	
IV	0 (0%)	0 (0%)	
Extra-intestinal CD manifestations [yes/no]	1 (5.9%):16 (94.1%)	14 (11.3%):110 (88.7%)	0.69 ^#^
Duration of CD [years]	8.6 ± 6.8	10.6 ± 9.0	0.37 *
Duration of CD > 10 years [yes/no]	7 (41.1%):10 (58.9%)	51 (41.1%):73 (58.9%)	1.00 ^#^
Smoking	6 (35.5%)	35 (28.2%)	0.58 ^#^
Steroids > 20 mg/d	6 (35.3%)	22 (17.7%)	0.11 ^#^
Anti-TNF-α 4–8 weeks prior to surgery	3 (17.6%)	28 (22.6%)	0.76 ^#^
Azathioprine/5-MCP	5 (29.4%)	41 (33.1%)	1.00 ^#^
Other immunomodulators	1 (5.9%)	3 (2.4%)	0.41 ^#^
>1 Immunomodulator	5 (29.4%)	36 (29.0%)	1.00 ^#^
Anticoagulation			1.00 ^#^
None	17 (100%)	123 (99.2%)	
ASA	0 (0%)	1 (0.8%)	
Previous abdominal surgery			0.79 ^#^
None	7 (41.2%)	60 (48.4%)	
Minor	3 (17.6%)	23 (18.5%)	
Major	7 (41.2%)	41 (33.1%)	
Pre-existing intra-abdominal sepsis	10 (58.8%)	45 (36.3%)	0.11 ^#^
Pre-existing intra-abdominal fistula	9 (52.9%)	38 (30.6%)	0.097 ^#^
Pre-existing intra-abdominal abscess	8 (47.1%)	18 (14.5%)	**0.004 ^#^**
Liver disease	0 (0%)	3 (2.4%)	1.00 ^#^
Diabetes mellitus	1 (5.9%)	1 (0.8%)	0.23 ^#^
CRP [mg/L] **	14.5 ± 8.2	5.1 ± 7.2	0.035 *
Leukocyte count [1000/µL] **	10.3 ± 5.3	8.8 ± 4.0	0.30 *
Hemoglobin level [g/dL] **	11.4 ± 1.7	12.5 ± 2.0	0.027 *
Type of operation			0.51 ^#^
Right hemicolectomy	3 (17.6%)	9 (7.3%)	
Ileocecal resection	11 (64.7%)	87 (70.2%)	
Anastomotic resection	3 (17.6%)	26 (21.0%)	
Restoration of continuity	0 (0%)	2 (1.6%)	
Access to the abdomen			0.08 ^#^
Laparoscopic	5 (29.4%)	65 (52.4%)	
Open	12 (70.6%)	51 (41.1%)	
Type of primary surgeon			0.14 ^#^
Resident	7 (41.2%)	29 (23.4%)	
Consultant	10 (58.8%)	85 (68.5%)	
Stapled:handsewn	8 (47.1%):9 (52.9%)	70 (56.5%):54 (43.5%)	0.60 ^#^
Type of anastomosis			0.81 ^#^
End-to-end	7 (41.2%)	44 (35.5%)	
Side-to-side	10 (58.8%)	79 (63.7%)	
End-to-side	0 (0%)	1 (0.8%)	
Side-to-end	0 (0%)	0 (0%)	

* *t*-test; ^#^ Fisher’s exact test; ** laboratory parameter on the day of admission; 5-MCP: 5-mercaptopurine; ASA: acetylsalicylic acid; anti-TNF-α: anti-tumor necrosis factor-alpha therapy; ASA classification: American Society of Anesthesiologists (ASA) Physical Status classification system; BMI: body mass index; CCI: Charlson comorbidity index; CD: Crohn’s disease; CRP: C-reactive protein.

**Table 7:** Final logistic regression model after AIC-based forward variable selection for the subgroup of *n* = 141 Crohn’s disease patients. *p*-values are conditional on the final model specification.

	OR *	95% CI **	*p*-Value
Type of surgeon			
Resident	—	—	
Consultant	0.418	0.04–0.72	**0.018**
Pre-existing intra-abdominal abscess	7.19	1.63–35.16	**0.010**
Indications for elective surgery			
All other diagnoses	—	—	
Anastomotic resection of previous ileocolic anastomosis for surgical recurrence	0.96	0.15–5.26	0.963
Penetrating disease with intra-abdominal abscesses or blind-ending fistulas	0.10	0.004–0.863	0.071
Access to the abdomen			
Laparoscopic or converted	—	—	
Open	4.09	1.01–19.14	0.056
Age [years]	0.94	0.87–1.00	0.076
BMI [kg/m^2^]	1.15	1.004–1.340	0.049

* OR = odds ratio; ** CI = confidence interval; BMI: body mass index.

## Data Availability

The data used to support the findings of this study are included in the article (Table 1, Table 2, Table 3, Table 4, Table 5, Table 6 and Table 7). Further information can be requested from the corresponding author.
